# Supplementary material for: Chip-scale sensor for spectroscopic metrology
Source: Nat Commun. 2024 Nov 27;15:10305. doi: 10.1038/s41467-024-54708-x (PMC11603224; doi:10.1038/s41467-024-54708-x)
Supplement: Supplementary file 1 — Supplementary Information [file 41467_2024_54708_MOESM1_ESM.pdf]

Supplementary Information

Chip-scale sensor for spectroscopic metrology

Chunhui Yao[1,2]†, Wanlu Zhang[1]†, Peng Bao[1], Jie Ma[2], Wei Zhuo[2], Minjia Chen[1], Zhitian Shi[1], Jingwen Zhou[2], Yuxiao Ye[2], Liang Ming[2], Ting Yan[2], Richard Penty[1], Qixiang Cheng[1,2*]

1. Electrical Engineering Division, Department of Engineering, University of Cambridge, UK
2. GlitterinTech Limited, Xuzhou, China

*qc223@cam.ac.uk;

†These two authors contribute equally to this work.

Section 1 Complete sampling matrix

Figure S1 presents the complete sampling matrix with 4096 channels, showing consistent pseudo-random spectral fluctuations over a 520 nm wavelength range. Due to the bandwidth constraints of our SLD sources and the limited measurement range of the benchtop optical spectrum analyser, the calibration of sampling matrix cut off at 1700 nm. We expect the actual operational bandwidth of our RS to exceed 1700 nm.

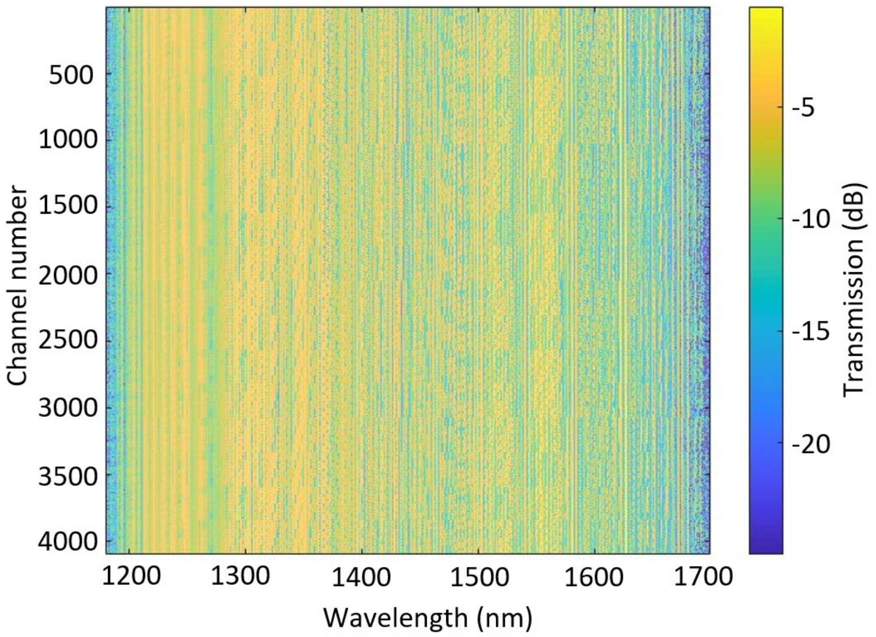

Figure S1. Normalized sampling matrix between 1180 nm and 1700 nm, including the entire 4096 channels

Section 2 Additional investigation on RS resolution

To verify the consistency of the spectrometer's resolution across the entire bandwidth, we rigorously simulated the dual-peak test (i.e., following the Rayleigh criterion) at different wavelength regions, as shown in Fig. S2. All simulations accounted for an equivalent level of measurement noise observed during our experiments.

Figure S2a-c depict the reconstructed dual-peak signals with 8 pm spacing located at about

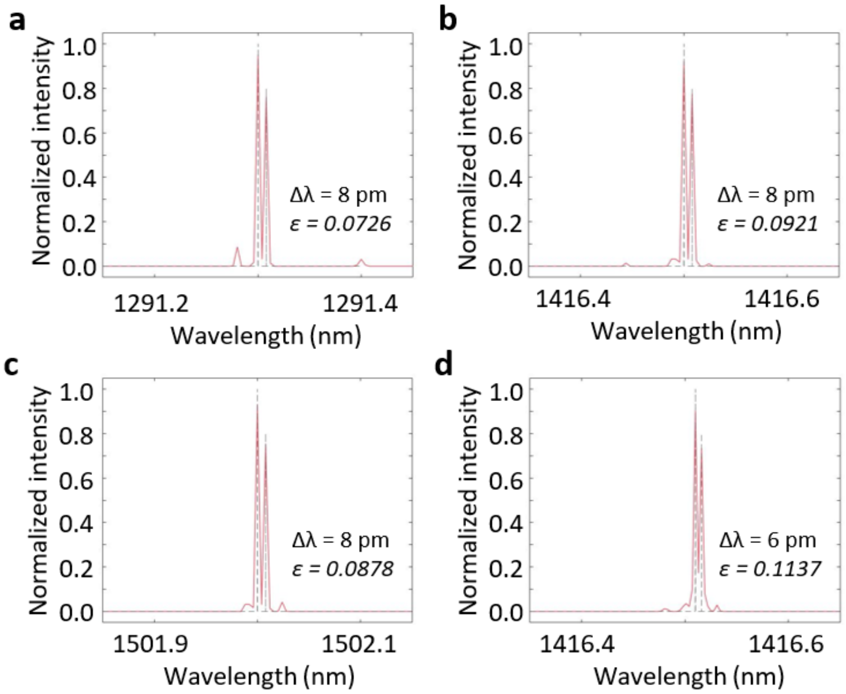

Figure S2. Simulated reconstruction of dual-peak signals at different wavelengths. (a-c) reconstructed dual-peak signals with 8 pm spectral spacing, located at around 1291 nm, 1416 nm, and 1502 nm, respectively. (d) Reconstructed dual-peak signal with 6 pm spectral spacing located at around 1416 nm.

1291 nm, 1416 nm, and 1502 nm, respectively. The low relative errors, ranging from around 0.073 to 0.088, illustrate the resolution consistency of our RS across its bandwidth. Additionally, considering the denser spectral fluctuations at shorter wavelengths (see Fig. 3C), we further reduced the spectral spacing to 6 pm for a dual-peak signal at 1416 nm. As shown in Fig. S2d, a relative error of 0.11 is achieved, indicating a slight improvement in resolution at shorter wavelengths.

Section 3 Thermal stability

Temperature stability is a crucial metric for on-chip spectrometers, as any variations can distort the channel spectral responses. Hence, we choose to implement our RS on a SiN platform to leverage its low thermal sensitivity. To test the device's thermal stability, we repeatedly record the spectral response of a same channel under different ambient temperature conditions ranging from 10°C to 30°C in 2°C increments. As shown in Fig. S3a-d, the channel response only redshifts by 0.32 nm when the temperature increases from 10 °C to 30 °C, while the waveform remains unchanged.

Accordingly, we model the RS's sampling matrices under various temperatures and calculated the corresponding output power intensities at all sampling channels using narrowband laser signals at different wavelengths as inputs. Using these power intensities, we then proceed with spectrum reconstructions based on the sampling matrix obtained under no temperature variation. As shown in Fig. S3e,f, even with temperature variations of up to ±0.75°C, the input laser signals can still be recovered accurately within the spectrometer's

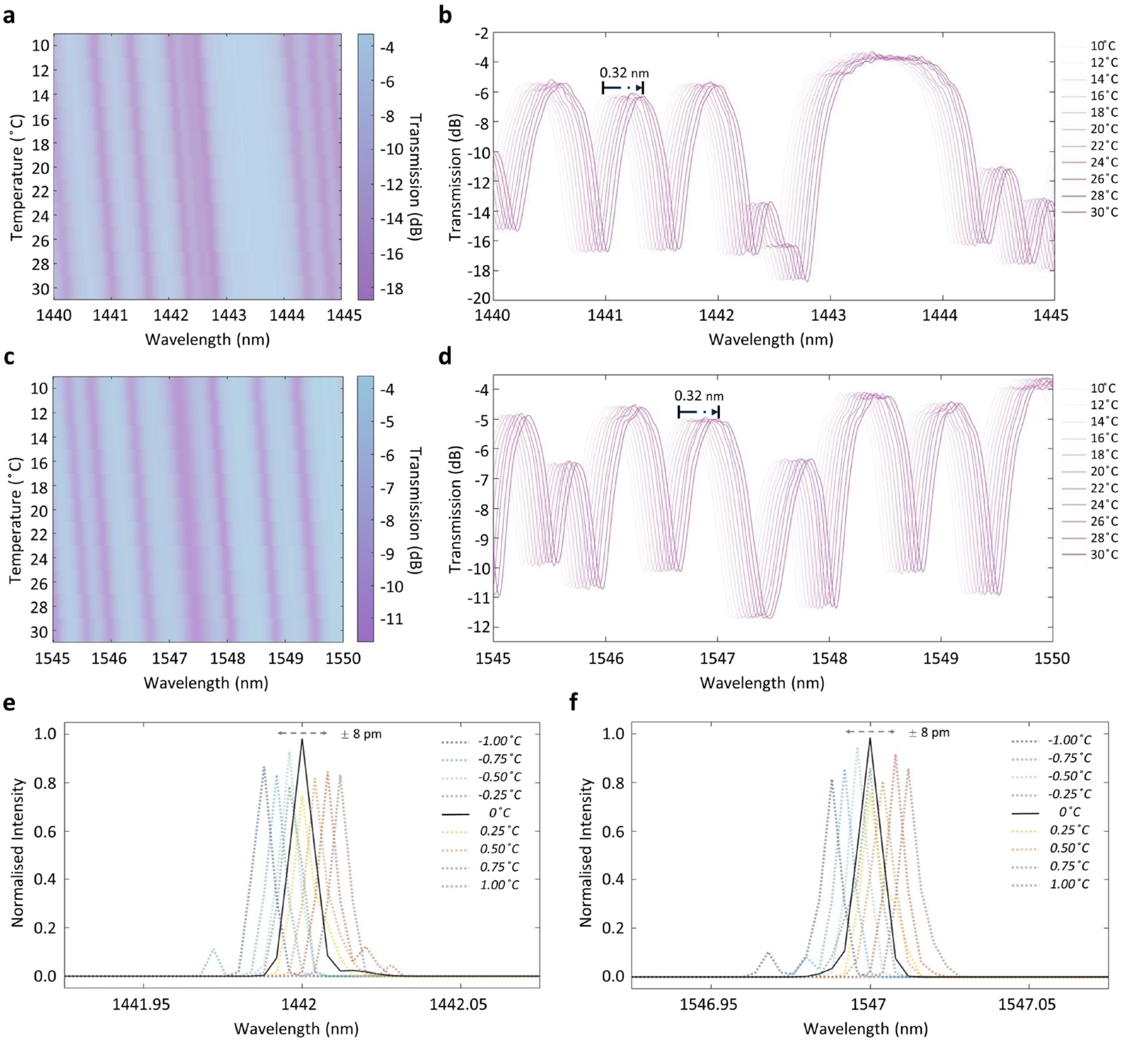

Figure S3. Thermal stability of our RS. (a,b) The channel spectral responses within an observation window between 1400 nm and 1445 nm under varying temperatures from 10°C to 30°C, plotted in matrix format and as a discrete line graph, respectively. (c,d) The channel spectral responses within an observation window between 1545 nm to 1550 nm, plotted in matrix format and as a discrete line graph, respectively. (e,f) Reconstructed laser signals at 1443 nm and 1548 nm under different temperature variations, showing good tolerance to temperature variations up to ± 0.75 °C.

resolution, with the center wavelength offset remaining within ±8 pm.

In practice, potential temperature drift can be mitigated by various stabilization techniques, while real-time temperature monitoring can also help offset the waveform shifts from a software perspective.

Section 4 Reflectance spectra of plastic and coffee samples

Figure S4a,b present the reflectance spectra of the remaining eight plastic and coffee samples, respectively, showing that our sensor maintains excellent consistency during repeated measurements. In Fig. S4c,d, we summarize the reflectance spectra for all ten types of plastic and coffee samples within specific observation windows, respectively. Subtle differences in NIR spectral features can be observed among different samples. To better quantify the

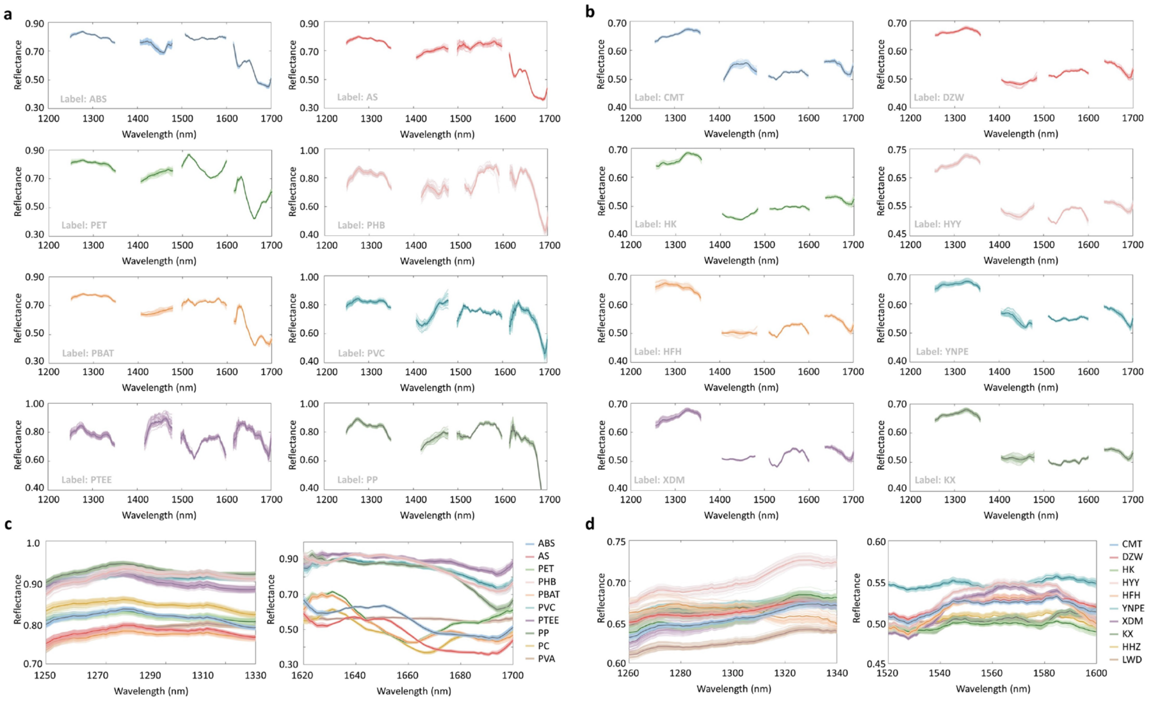

Figure S4. Reflectance spectra of plastic and coffee samples. (a,b) Measured reflectance spectra for the remaining plastic and coffee samples, respectively. (c,d) Measured reflectance spectra for all ten types of plastic and coffee samples, respectively, presented in different observation windows. Subtle variations in spectral features can be observed among different samples.

reconstruction accuracy, Table S1 outlines the average relative error and standard deviation from 60 tests on each plastic and coffee sample. As shown, the measured plastic and coffee samples exhibit average relative errors ranging from 0.026 to 0.054 and 0.031 to 0.056, respectively. Additionally, the calculated standard deviations for all samples remain around 0.02. These results demonstrate the excellent precision and stability of our sensor.

To investigate the relationship between model accuracy and spectral reconstruction error, we generate a series of spectra with varying levels of relative error by artificially superimposing

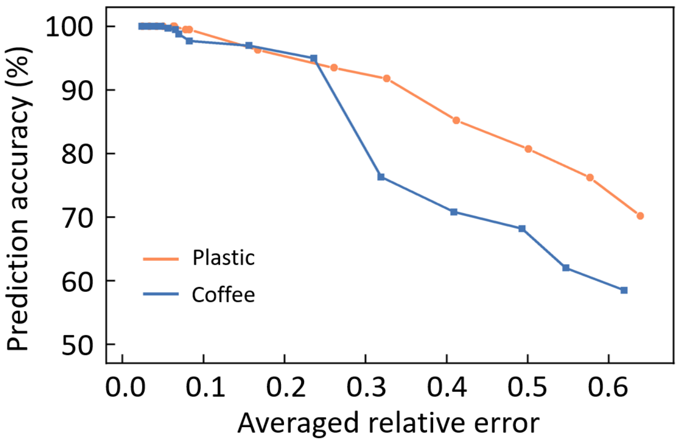

Figure S5. Relationship between the model prediction accuracy and spectral relative error for plastic and coffee samples.

Table S1. Average relative error and standard deviation of tests for plastic and coffee samples

Plastic sample	Mean	Standard deviation	Coffee sample	Mean	Standard deviation
ABS	0.0323	0.0236	CMT	0.0414	0.0206
AS	0.0537	0.0266	DZW	0.0376	0.0247
PBAT	0.0463	0.0260	HFH	0.0429	0.0228
PET	0.0378	0.0253	HK	0.0317	0.0269
PHB	0.0457	0.0214	HYY	0.0488	0.0215
PP	0.0261	0.0182	KX	0.0305	0.0268
PTEE	0.0530	0.0314	XDM	0.0473	0.0291
PVC	0.0540	0.0241	YNPE	0.0524	0.0257
PC	0.0373	0.0154	HHZ	0.0455	0.0204
PVA	0.0424	0.0168	LWD	0.0560	0.0195

random, continuous spectral errors of different magnitudes onto the reference spectra of each sample. Following the same training process, we then evaluate the model's prediction accuracy under different levels of relative error, as shown in Fig. S5. The results indicate that for plastic samples, model accuracy begins to drop from 100% when the average relative error reaches approximately 0.08, decreasing to 70% at a relative error of around 0.64. Similarly, for coffee samples, accuracy can no longer be maintained at 100% once the relative error exceeds about 0.06, dropping to around 60% when the relative error surpasses 0.55.

Section 5 Performance comparison with benchtop dispersive and FT spectrometers

For performance comparison, the identical glucose concentration test is also performed using a benchtop dispersive spectrometer (IdeaOptics NIR17+Px), and a benchtop Fourier transform spectrometer (Bruker MPA II). Here, we include an additional 0.05% glucose solution to validate their detection limits. For modelling, we adopt the same random forest prediction model as described in the main text, and utilize the measured spectra data from both instruments across their full operational bandwidths as well as the selected wavelength range between 1180 nm and 1700 nm. Figure S6a-d show the performance of the prediction models for each instrument under different wavelength ranges. When using the full bandwidth, the

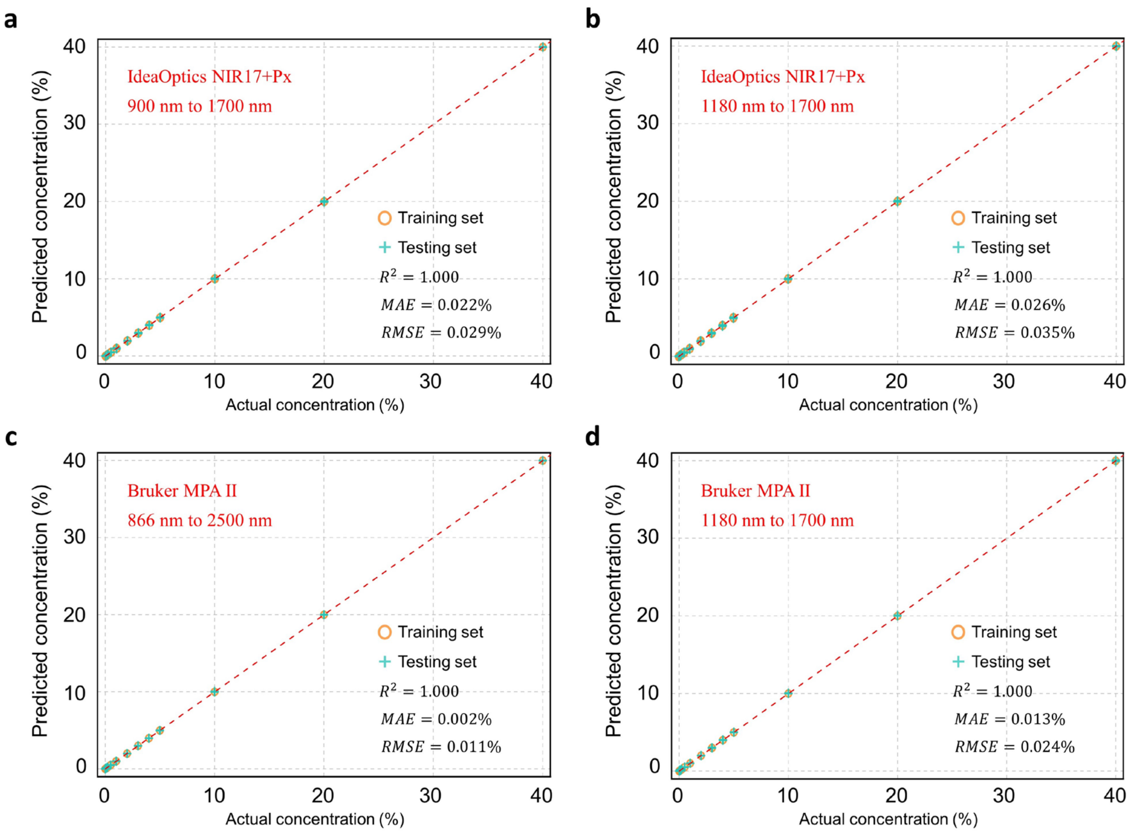

Figure S6. Concentration test of glucose solutions using commercial benchtop spectrometers. (a, b) Predicted glucose concentrations using the full and partial bandwidths of a dispersive spectrometer (IdeaOptics NIR17+Px), respectively. (c, d) Predicted glucose concentrations using the full and partial bandwidths of a FT spectrometer (Bruker MPA II), respectively.

IdeaOptics NIR17+Px achieves a detection limit of 0.1%, with the MAE and RMSE being 0.022% and 0.029%, respectively; while the Bruker MPA II realizes a detection limit of 0.05% with lower MAE and RMSE values. When the modelling range is confined to 1180 nm to 1700 nm, both instruments have a detection limit of 0.1%, with increased MAE and RMSE values to 0.026% and 0.013%, and 0.035% and 0.024%, respectively.

These results indicate that our sensor accomplishes comparable detection limits and prediction errors to the benchtop competitors. Notably, leveraging the scalability of our design, the sensor's bandwidth can be readily extended by co-packaging multiple chiplets, each operating in different wavelength ranges. This can, thereby, further improve the detection limit and prediction accuracy of our NIR sensor.

Section 6 Design details of Curved DCs

Following the design criteria described in the main text, this section further elaborates on the geometric design of a curved DC targeted at the wavelength range between 700 nm and 1200 nm, demonstrating the wavelength scalability of our design. The optimization goal is to ensure that the MRR maintains the desired over-coupling state across the entire wavelength range to match the increasing round-trip loss in the ring. The PSO algorithm is employed to

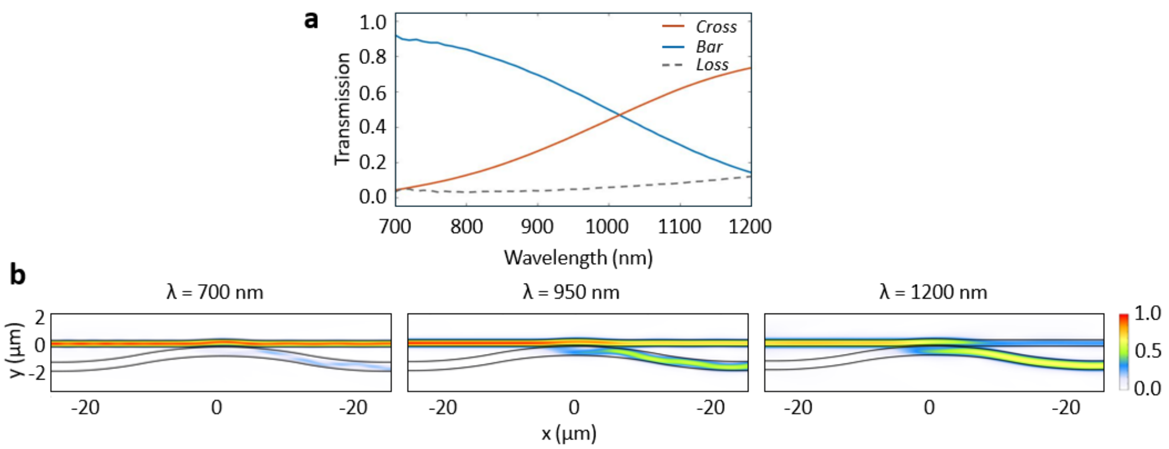

Figure S7. Optimized curved DC design targeted between 700 nm and 1200 nm. (a) Simulated coupling efficiency among the entire waveband. (b) Simulated light propagation profiles (i.e. electric field intensity) in the curved DC at different wavelengths.

optimize all the structure parameters, including the waveguide widths ($\boldsymbol{W_{bus}}$ and $\boldsymbol{W_{ring}}$), gap ($\boldsymbol{G}$), radius ($\boldsymbol{R_1}$), and the curvature angle ($\boldsymbol{\theta}$). Figure S7a,b present the FDTD-simulated coupling efficiency and light propagation profiles of the optimized curved DC, respectively, showing the increasing cross coupling efficiency over wavelength.

In summary, Table S2 lists the optimized design parameters for the curved DC designs with different working bandwidths between 700 nm to 1200 nm, and 1200 nm to 1700 nm, respectively.

Table S2. Geometric parameters for the curved DC designs

**Working bandwidth**	**Bus width** W_{bus}	**Ring width** W_{ring}	**Gap** G	**Inner radius** R_1	**Curvature angle** θ
700 – 1200 nm	0.6 µm	0.85 µm	0.12 µm	23 µm	8°
1200 – 1700 nm	1.1 µm	1.15 µm	0.30 µm	60 µm	18.1°

Section 7 Optical testbed and sampling interfaces

Figure S8 illustrates the optical testbed used for the calibration of RS, reconstructions of unknown incident spectra, and NIR spectrometric sensing applications. During calibration, four SLDs with different center wavelengths are sequentially introduced to the RS chip, and an optical spectral analyzer (YOKAGAWA AQ6370D) measures the spectral responses of different temporal sampling channels. For resolving various unknown spectra using the RS chip, the received signals are monitored by an optical power meter, which tracks the aggregated power after the spectra propagate through different sampling channels. The measured power

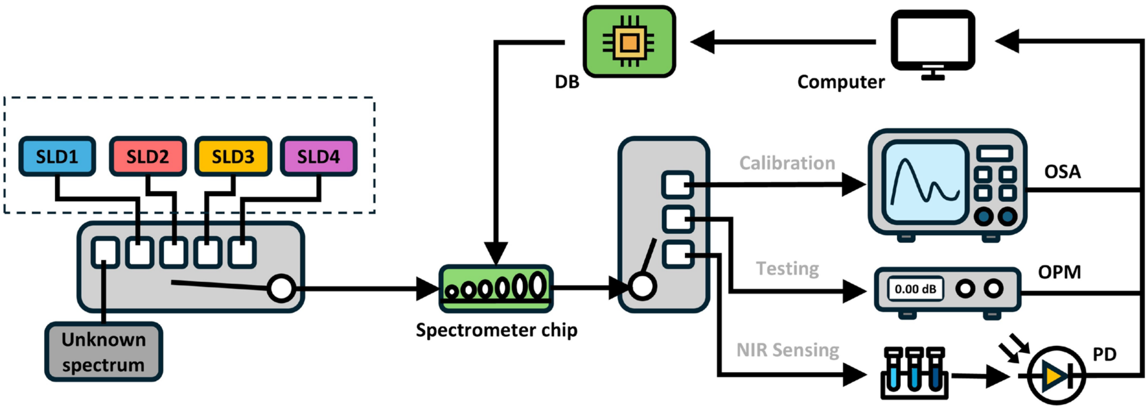

Figure S8. Schematic of the optical testbed for calibration, unknown spectra reconstruction, and NIR spectrometric sensing applications. DB: driving board. OSA: optical spectrum analyzer. OPM: optical power meter. PD: photodiode.

intensities are then used for computational reconstruction. In NIR spectrometric sensing for various substances, the four SLDs are also sequentially activated to illuminate the samples under test. The reflected or transmitted light from the sample is collected by photodiodes (PDs). It should be noted that there are some low-power regions between different SLD sources (see the inset in Fig. 4d), leading to minor discontinuities in our NIR spectral measurements. This can be readily addressed by implementing more SLDs with different operational wavelength ranges or using SLDs with higher power and broader bandwidths.

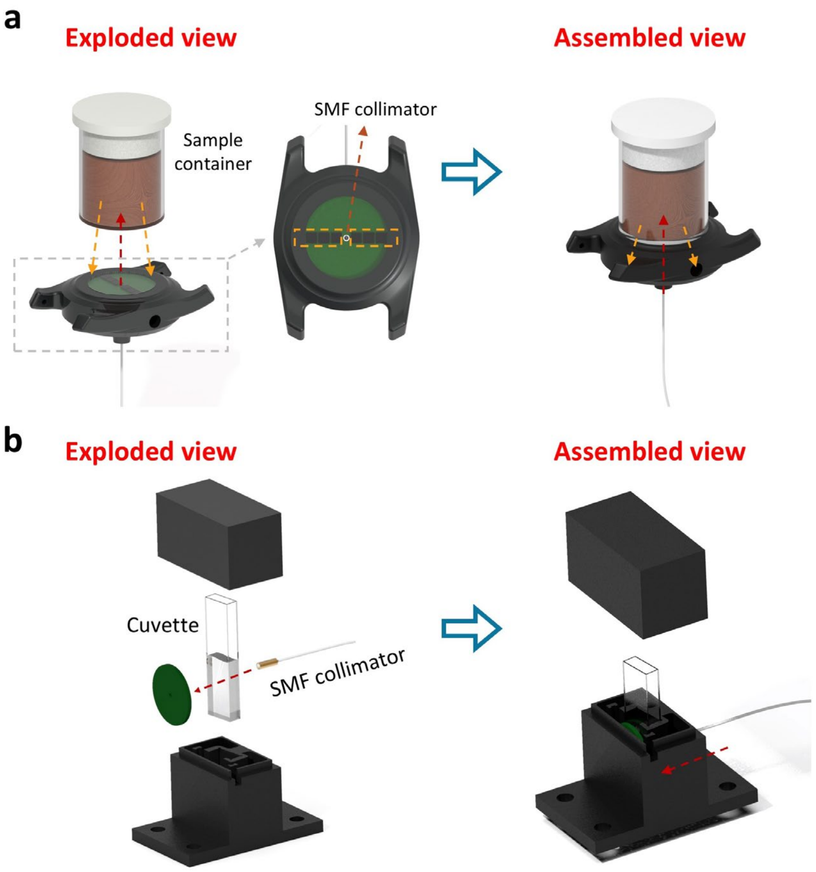

Figure S9. (a, b) Optical sampling interfaces for solid and liquid samples, respectively, presented in both exploded and assembled views.

To efficiently measure the reflection or transmission spectra from various samples, two free-space optical sampling interfaces are developed. Figure S9a shows the sampling interface designed for solid substances, which includes a single-mode fiber (SMF) collimator surrounded by six 2 mm photodiodes (PDs), three on each side. A glass sample container is placed directly above this interface, allowing the illuminating SLD sources to reach the sample and reflect back to the PDs, thereby capturing the reflectance information. Figure S9b depicts the sampling interface for liquid samples, consisting of an SMF, a cuvette, and surface PDs. In this setup, the light beam generated by the SLDs passes through the liquid sample in the cuvette and is collected by the surface PDs. Consequently, the measured optical power intensities are used to reconstruct the sample's transmission spectra.
